# Retron reverse transcriptase termination and phage defense are dependent on host RNase H1

**DOI:** 10.1093/nar/gkac177

**Published:** 2022-03-16

**Authors:** Christina Palka, Chloe B Fishman, Santi Bhattarai-Kline, Samuel A Myers, Seth L Shipman

**Affiliations:** Gladstone Institute of Data Science and Biotechnology, San Francisco, CA, USA; Gladstone Institute of Data Science and Biotechnology, San Francisco, CA, USA; Gladstone Institute of Data Science and Biotechnology, San Francisco, CA, USA; La Jolla Institute for Immunology, La Jolla, CA, USA; Gladstone Institute of Data Science and Biotechnology, San Francisco, CA, USA; Department of Bioengineering and Therapeutic Sciences, University of California, San Francisco, CA, USA

## Abstract

Retrons are bacterial retroelements that produce single-stranded, reverse-transcribed DNA (RT-DNA) that is a critical part of a newly discovered phage defense system. Short retron RT-DNAs are produced from larger, structured RNAs via a unique 2′-5′ initiation and a mechanism for precise termination that is not yet understood. Interestingly, retron reverse transcriptases (RTs) typically lack an RNase H domain and, therefore, depend on endogenous RNase H1 to remove RNA templates from RT-DNA. We find evidence for an expanded role of RNase H1 in the mechanism of RT-DNA termination, beyond the mere removal of RNA from RT-DNA:RNA hybrids. We show that endogenous RNase H1 determines the termination point of the retron RT-DNA, with differing effects across retron subtypes, and that these effects can be recapitulated using a reduced, *in vitro* system. We exclude mechanisms of termination that rely on steric effects of RNase H1 or RNA secondary structure and, instead, propose a model in which the tertiary structure of the single-stranded RT-DNA and remaining RNA template results in termination. Finally, we show that this mechanism affects cellular function, as retron-based phage defense is weaker in the absence of RNase H1.

## INTRODUCTION

Retrons are prokaryotic retroelements that have recently been shown to play a role in phage defense (1–4). Retrons consist of three components: (1) a reverse transcriptase (RT), (2) a structured non-coding RNA (ncRNA) that is reverse transcribed by the RT into single-stranded, reverse-transcribed DNA (RT-DNA), and (3) and one or more accessory proteins. Bioinformatic analyses based on sequence have identified more than 2,000 proposed retrons, accounting for up to ∼25% of all bacterial retroelements, though to date fewer than twenty have been verified and characterized biochemically (5–8). Despite having only a cursory understanding of retron mechanism and function, this RT family has generated substantial interest for its ability to produce single-stranded RT-DNA *in vivo*, which allows for synthetic biology applications in eukaryotic gene editing (9), recombineering (10) and high throughput mutagenesis (11).

**Figure 1.**
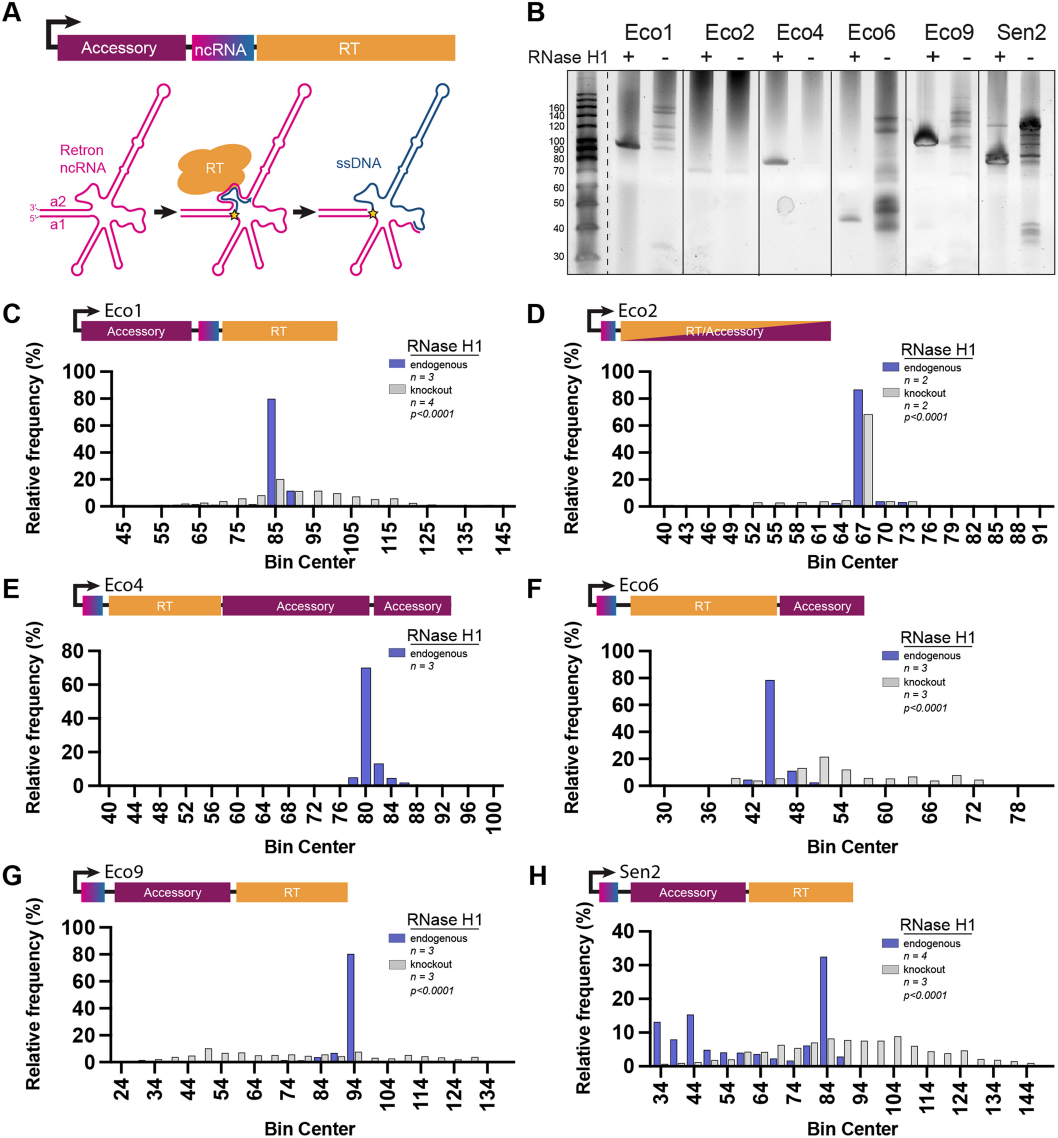
Endogenous RNase H1 determines retron RT-DNA length. (**A**) Schematic showing retron RT-DNA synthesis and the retron operon of retron-Eco1. The ncRNA is pink, the RT-DNA is blue, the accessory protein is maroon and the RT is orange. The star represents the 2′-5′ linkage. (**B**) A TBE–urea polyacrylamide gel showing RT-DNA production across six retrons in the presence and absence of RNase H1. (**C–H**) RT-DNA length quantification, determined by multiplexed sequencing. Samples containing retron sequence were analyzed to determine length and binned into histograms. Purple bars are RT-DNA produced in the presence of RNase H1, gray bars are RT-DNA produced in the absence of RNase H1, p values are a Mann Whitney test of the length distribution. Statistical details in Supplementary Table S1.

Retrons employ a unique mechanism for RT-DNA synthesis. The ncRNA template folds into a conserved secondary structure, insulated between two inverted repeats (a1/a2, Figure 1A). The retron RT recognizes the folded ncRNA and reverse transcription is initiated from a conserved guanosine 2′-OH adjacent to the inverted repeats, forming a 2′-5′ linkage between the template RNA and the nascent DNA strand (12–15). In some retrons this 2′-5′ linkage persists into the mature form of processed RT-DNA (16), while in others an exonuclease cleaves the DNA product resulting in a free 5′ end (17,18).

Unlike viral reverse transcriptases, retron RTs do not fully reverse transcribe their template RNAs as a means of replication, but instead terminate at a precise point in the middle of the retron ncRNA (Figure 1A). This results in a short RT-DNA product that varies in size by subtype from ∼40–160 bases. When isolated from cells, retron RT-DNA is single stranded and degradation of the RNA template has been shown to depend on endogenous RNase H1 (19). Prior experiments suggest that RNase H1 cleavage occurs quickly or perhaps is even coupled with reverse transcription (19). In another study it was observed that in the absence of RNase H1 the length of Eco5 RT-DNA was altered, but in the same conditions the length of Eco7 was unaffected (15,19). The mechanism by which retrons terminate reverse transcription is not well understood.

Recent work describes a function for the retron as a cellular defense system against phages (1–4), in which the retron RT-DNA may act as a phage sensor. In this model, phage infection activates a toxic effect of the accessory protein that results in abortive infection, which depends on the synthesis of RT-DNA and 2–5′ linkage processing (in retrons where that processing occurs) (1–4). Multiple components of this model remain mysterious, including whether or how the RT-DNA neutralizes the toxic accessory protein in the absence of phage, and releases or activates it in the presence of phage. These mechanisms remain poorly understood in part because we do not understand the properties of the RT-DNA itself, warranting further study of the synthesis of the retron RT-DNA.

Experiments suggest that retron RNA is cleaved by RNase H as RT-DNA is synthesized, rather than after synthesis is complete (19), demonstrating a close relationship between RNase H1 and the retron complex. This close association, combined with the importance of RT-DNA in phage defense, prompted us to test whether RNase H1 plays a role in RT-DNA synthesis beyond simply removing the RNA in the DNA:RNA hybrid. We extend previous observations (15,19), finding a prominent effect of host RNase H1 on RT-DNA termination, both *in vivo* and in a reduced *in vitro* system. Using this as a starting point to investigate retron RT-DNA termination, we find that, in addition to RNase H1, precise termination requires the native architecture of the ncRNA. We propose a model in which removal of the RNA template leads to a tertiary DNA:RNA structure that enforces termination. At a cellular level, we show that RNase H1 is also critical for retron-based phage defense.

## MATERIALS AND METHODS

All biological replicates were collected from distinct samples and not the same sample measured repeatedly.

### Strain generation

Bacterial experiments were carried out in BL21-AI cells, or a derivative of BL21-AI cells. These cells harbor a T7 polymerase driven by an arabinose-inducible promoter. A knockout strain for the Eco1 operon (BL21*^Δ^^Eco1^*) was previously described (20). An additional strain (BL21*^Δ^^Eco1;^^Δ^^rnhA^*) was constructed from that strain, using a strategy based on Datsenko and Wanner (21) to replace the rnhA operon with an FRT-flanked chloramphenicol resistance cassette. The replacement cassette was amplified from pKD3, adding homology arms to the rnhA locus. This amplicon was electroporated into BL21-AI cells expressing lambda Red genes from pKD46 and clones were isolated by selection on 10 μg/ml chloramphenicol plates. After genotyping to confirm locus-specific insertion, the chloramphenicol cassette was excised by transient expression of FLP recombinase to leave only an FRT scar. Strain genotypes are listed in Supplementary Table S3.

### RT-DNA expression, purification and PAGE gel analysis

Constructs (Supplementary Table S2) were transformed via electroporation into either strain BL21*^Δ^^Eco1^ or* BL21*^Δ^^Eco1;^^Δ^^rnhA^*. Selection for successfully transformed cells was performed by growing the transformed cultures on kanamycin plates. A single colony from the culture was picked and grown in 5 ml of LB and 1 mM kanamycin, shaking overnight at 37°C. The following morning 100 ul of the overnight culture was inoculated into 5 ml of LB and 1 mM kanamycin and allowed to shake at 37C for 2 h during which the cells entered log growth phase. The full 5mL culture was then added to 50 ml of LB, 35 ug/ml kanamycin, 1 mM IPTG and 200 ug/ml l-arabinose. Cells were allowed to grow for 5–8 h after which the cells were pelleted and stored at –20°C.

To visualize RT-DNA on a PAGE gel the cell cultures were prepared using a Qiagen plasmid plus midi prep protocol and eluted in a volume of 100 ul. 5 ul of the purified RT-DNA was then analyzed on 10% Novex TBE–urea Gels (Invitrogen), with a 1× TBE running buffer that was heated to ∼50°C before loading. Gels were stained with Sybr Gold (Thermo Fisher) and imaged on a Gel Doc imager (Bio-Rad).

### Illumina multiplexed DNA sequencing

After the Qiagen midiprep, 86 ul of the RT-DNA was treated with 10 units of RNase H1 (NEB) and ∼20 ng of DBR1 (Origene or in-house prep, see below) in 1× Cutsmart for 30 min at 37°C and then single stranded DNA was isolated from the prep using an ssDNA/RNA Clean & Concentrator (Zymo Research) and eluted in a volume of 16 ul.

RT-DNA was prepped for sequencing by taking 9 ul of the ssDNA/RNA cleanup and extending the 3′ end with a single nucleotide, dATP, in a reaction with terminal deoxynucleotidyl transferase (TdT) (NEB). This reaction was carried out in 1× TdT buffer, with 60 units of TdT and 125 μM dATP for 60 s at room temperature with the aim of adding ∼25 adenosines before inactivating the TdT at 70°C for 5 min. 8 ul of the TdT extended product was carried forward and a second complementary strand was then created from that extended product using 15 units of Klenow Fragment (3′→5′ exo-) (NEB) in 1× NEB2, 1 mM dNTP and 50 nM of primer containing an Illumina adapter sequence, nine thymines, and a non-thymine (V) anchor. Product was cleaned up using Qiagen PCR cleanup kit and eluted in 25 ul water. Finally, Illumina adapters were ligated on at the 3′ end of the complementary strand using 1× TA Ligase Master Mix (NEB). All products were indexed and sequenced on an Illumina MiSeq instrument. All samples were analyzed in at least two biological replicates (different transformations) with exact N indicated on the figure and in Supplementary Table S1. Primers used for sequencing are listed in Supplementary Table S4.

### Sequencing analysis

Sequencing reads were analyzed using custom Python software written to trim Illumina adaptors and poly-A tails. Three 10-base sequences present along the RT-DNA, specific to each retron, was queried, allowing for one mismatch. All sequences containing at least one of those sequences were analyzed for length.

For length analysis, the total number of reads that were counted for each length was tallied and then normalized to the total number of reads that contained any RT-DNA sequence (reads per length/total RT-DNA reads). Length values were then analyzed for relative frequency and plotted into a binned histogram using Prism software.

To analyze the sequence that was reverse transcribed, retron RT-DNA containing reads were exported as a FASTA file and assembled in Geneious to a sequence starting at the T7 promoter and extending to the start codon of the retron-RT. Assembled reads were exported as a .csv file and % coverage was calculated by dividing the reads at each nucleotide by the total number of reads that contained RT-DNA.

To measure terminal base bias of the sequencing approach, four oligonucleotides were combined in equal parts to a total concentration of 1 μM. This mixture was prepped for sequencing with the same method as the retron-derived RT-DNA. Sequencing reads were analyzed with a custom Python script to identify the oligo sequence of interest. The full oligonucleotide sequence was queried using fuzzysearch, allowing for two mismatches including the undefined terminal base. Each read that matched the oligo sequence was then binned according to its terminal base.

### *In vitro* transcription of Eco1 RNA and Eco1 chemical mapping RNA

RNA template was generated using PCR assembly by primers designed using Primerize (22,23). RNAs were in vitro transcribed using T7 RNA polymerase (NEB) in RNA polymerase reaction buffer (40 mM Tris–HCl, pH 7.9, 28 mM MgCl_2_, 90 mM DTT, 2 mM spermidine, 1.5 mM each NTP and 40 U RNasin Plus Ribonuclease Inhibitor [Promega]). The reaction was incubated at 37°C for 3 hours followed by the addition of 10 units of TURBO DNase (Thermo Fisher) for 15 min at 37°C. RNA was purified using a Zymo RNA clean and concentrate kit.

### Eco1-RT and DBR1 cloning and expression

Eco1-RT was cloned into the pSol-Tsf backbone from Lucigen's kit - Expresso Solubility and Expression Screen. DBR1 was also cloned into the backbone but with an additional His-tag between the TEV site and DBR1. Constructs were transformed into BL21-AI cells and plated on an LB-kanamycin plate. A single colony was picked and grown overnight shaking at 37°C in 5 ml of LB with 1 mM Kanamycin. 5 ml of culture was added to 1 L of LB and allowed to reach an OD of 0.5 shaking at 250 rpm in a baffled flask at 37°C. For Eco1-RT, the culture was then placed on ice for 15 min. 1% arabinose and 0.02% l-rhamnose (1 ml of 20% solution) were added to induce expression and culture was allowed to shake at 18°C for ∼18 h. DBR1 was induced with 1% Arabinose and 0.02% l-rhaminose (1 ml of 20% solution) and culture was allowed to shake at 37°C for 4 h. Cells were then spun down and stored at –20°C or moved directly into the protein purification protocol below.

Eco1 RT and DBR1 purification was achieved by resuspending the 1 L cell pellet in 30 ml of lysis buffer (20 mM Tris pH 7.4, 200 mM NaCl, 1 mM MgCl_2_, 10% glycerol). Cells were lysed once using the Misonix Sonicator 3000 on power level 5.0 with 30 s on/30 s off for 10 cycles. The lysed suspension was then spun at 18,000 rcf in a Beckman JL25.50 rotor at 4°C for 30 min. Supernatant volume was brought up to 50 ml with lysis buffer and 2 ml of equilibrated nickel excel resin (Cytiva) and rotated at 4°C for 3 h. Resin was then collected in 10 ml columns and washed using 25 ml of wash buffer (20 mM Tris pH 7.4, 200 mM NaCl, 1 mM MgCl_2_, 40 mM Imidazole pH 7.4 and 10% glycerol). Protein was eluted using 6 ml of elution buffer (20 mM Tris pH 7.4, 200 mM NaCl, 1 mM MgCl_2_, 300 mM Imidazole pH 7.4 and 10% glycerol). Protein was concentrated using an Amicon filter (10 kDa molecular weight cutoff) and purified using a Superdex200 (Cytiva) column on an AKTA Avant system. For Eco1-RT 280 UV wavelength was monitored and when protein elution started 100 ul fractions were taken. Fractions were run on a 4–12% gradient Bis–Tris NuPAGE gel in NuPAGE MES-SDS Running buffer. Only 100 ul fractions that contained minimal amount of co-purifying chaperone were taken. For DBR1, all peaks were run on a 4–12% gradient Bis–Tris NuPAGE gel in NuPAGE MES-SDS Running buffer and the fractions containing Dbr1 were taken. Protein size was approximated using a BLUEstain protein ladder (GoldBio). Gels were stained using Biotium One-Step Blue Protein Gel Stain. Proteins were aliquoted and stored at –20°C until use.

### *In vitro* functional assay for RT-DNA production

*In vitro* reactions contained 50 mM Tris–HCl (pH 7.8), 10 mM DTT, 10 mM MgCl_2_, 60 mM NaCl, 10% glycerol, 0.4 mM dNTP mix, 20 units of RNasin Plus Ribonuclease Inhibitor (Promega), 5 units of RNase H (NEB), 36 nM Eco1 ncRNA, and 3uM purified Eco1 Tsf-RT, unless otherwise indicated. Protein was added last. Reactions were allowed to incubate at 37°C for 1 h. Reactions were quenched with the addition of 5 units of RNase A/T1 (Thermo Scientific) and/or 30 units of RecJ (NEB) and/or DBR1 (Origene or in-house) by incubating at 37°C for 30 min. If RNase H1 was not included in the reaction, it was added with RNase A/T1 to remove DNA:RNA hybrids for PAGE visualization. After reaction was quenched, TBE–urea loading buffer was added to a 1× concentration. Samples were incubated at 90°C for 3 min and loaded into a 10% TBE–urea gel with 1× running buffer that had been heated to 55°C. Gels were run for ∼30 min, stained with SYBR-Gold and imaged.

### Chemical mapping

Chemical probing experiments were carried out as described previously (24–26). Briefly, 1.2 pmol of RNA was denaturated at 95°C in 50 mM Na-HEPES, pH 8.0, for 3 min, and folded by cooling to room temperature over 20 min and adding MgCl_2_ to the desired concentration of 10 mM. RNA was aliquoted in 15 μl volumes into a 96-well plate and mixed with nuclease-free H_2_O (control), or chemically modified in the presence of 5 mM 1-methyl-7-nitroisatoic anhydride (1M7) (27) for 10 min at room temperature. Chemical modification was stopped by adding 9.75 μl quench and purification mix (1.53 M NaCl, 1.5 μl washed oligo-dT beads, Ambion), 6.4 nM FAM-labeled, reverse-transcriptase primer, and 2.55 M Na-MES for 1M7. RNA in each well was purified by bead immobilization on a magnetic rack and two washes with 100 μl 70% ethanol. RNA was then resuspended in 2.5 μl nuclease-free water prior to reverse transcription.

RNA was reverse-transcribed from annealed fluorescent primer in a reaction containing 1 × First Strand Buffer (Thermo Fisher), 5 mM DTT, 0.8 mM dNTP mix, and 20 U of SuperScript III Reverse Transcriptase (Thermo Fisher) at 48°C for 30 min. RNA was hydrolyzed in the presence of 200 mM NaOH at 95°C for 3 min, then placed on ice for 3 min and quenched with 1 volume 5 M NaCl, 1 volume 2 M HCl, and 1 volume 3 M sodium acetate. cDNA was purified on magnetic beads as described previously, then eluted by incubation for 20 min in 11 μl Formamide-ROX350 mix (1000 μl Hi-Di Formamide [Thermo Fisher] and 8 μl ROX350 ladder [Thermo Fisher]). Samples were then transferred to a 96-well plate in ‘concentrated’ (4 μl sample + 11 μl ROX mix) and ‘dilute’ (1 μl sample + 14 μl ROX mix) for saturation correction in downstream analysis. Sample plates were sent to Elim Biopharmaceuticals for analysis by capillary electrophoresis.

### Chemical mapping analysis using HiTrace

Capillary electrophoresis runs from chemical probing were analyzed with the HiTRACE MATLAB package (28). Lanes of similar treatment groups (e.g. 1M7 modified) were aligned together, bands were fit to Gaussian peaks, background was subtracted using the no-modification lane, corrected for signal attenuation, and normalized to the internal hairpin control. The end result of these steps is a numerical array of ‘reactivity’ values for each RNA nucleotide. Three individual chemical mapping experiments were run to produce replicates.

### Mass spectrometry

Retron-RT constructs containing either a His-tag or a FLAG-tag on the C-terminus were created. Constructs were transformed into BL21-AI cells. 50 ml cultures of cells were grown to OD 0.6, cultures were cooled on ice, induced with 1mM IPTG, 1% arabinose, and expressed overnight shaking at 250 rpm at 18°C. After expression cultures were spun down, lysed, and soluble fraction isolated as described in the Tsf-RT expression method section. 30 ul of FLAG resin washed 4× with the wash buffer (20 mM Tris pH 7.4, 200 mM NaCl, 1 mM MgCl_2_, 40 mM Imidazole pH 7.4 and 10% glycerol) was added to 60 ul of the supernatant after high-speed spin. Beads and slurry were allowed to incubate for 4 h, rotating at 4°C, then were washed 3× with wash buffer and then 2× with wash II buffer (20 mM Tris pH 7.4, 200 mM NaCl, 1 mM MgCl_2_). Protein was eluted using cleavage of the TEV sequence between the tag and the RT. This was achieved by adding 10 units TEV (NEB) to the washed beads and incubating rotating overnight at 4°C. After cleavage beads were spun down and supernatant was collected and analyzed using mass spectrometry.

Samples were digested on-bead using LysC and trypsin overnight at 37°C without reduction or alkylation. Digests were desalted using Stage tips packed with C18 disks (EMPORE). After vacuum centrifugation, peptides were resuspended in 0.1% formic acid. Samples were analyzed on a Q-Exactive Plus mass spectrometer (Thermo) coupled to an Easy-nLC 1200 (Thermo). Data was processed using Spectrum Mill (Agilent Technologies) searching the Uniprot *Escherichia coli* B21 DE3 database (4430 entries, including common laboratory contaminants). Three missed cleavages were allowed, and variable modifications included N-term acetylation, methionine oxidation, pyro-glutamic acid, and deamidated asparagine. The mass tolerance for precursor and product ions was limited to 20 ppm.

### Accessory protein neutralization assay

Cultures of BL21*^Δ^^Eco1^* with the full retron operon or the respective accessory protein only were grown overnight. In the morning, cultures were diluted 1:5000 into 500 ul of LB and 1% kanamycin, with induced (1% l-arabinose and 1% IPTG) and uninduced conditions for each strain. Cultures were grown in 1 ml deep-well plates shaking vigorously at room temperature for 24 h. OD_600_ was measured in a molecular devices plate reader.

### Plaque assay

Phages were propagated from ATCC stocks (11303-B5, 11303-B2) into a liquid culture of *E. coli* (BL21*^Δ^^Eco1^*) at 37°C to OD600 0.3 in LB medium supplemented with 0.1 mM MnCl_2_ and 5 mM MgCl_2_ (MMB) until culture collapse, as described by (2,29,30). The culture was then centrifuged for 15 min at 6000 rpm and the supernatant was filtered through a 0.2 μm filter to remove bacterial remnants. Lysate titer was determined using the full plate plaque assay method as described in Kropinski *et al.* (31).

Small drop and full plate plaque assays were performed as previously described by Mazzocco *et al.* (32). Bacteria were grown overnight at 37°C. 60 ul of the overnight culture was passaged into 3 ml of MMB with kanamycin, l-arabinose and IPTG and grown for 2 h, shaking at 37°C.

For small drop plaque assays, 200 ul of the bacterial culture was mixed with 2 ml melted MMB agar (LB + 0.1 mM MnCl_2_ + 5 mM MgCl_2_ + 0.75% agar) and plated on MMB agar plates. The plates were dried for 20 min at room temperature. 10-fold serial dilutions in MMB were performed for each of the phages and 2 ul drops were placed on the bacterial layer.

Full plate plaque assays were set up on rectangular and circular plates, and the volume of bacterial culture added to create a lawn varied with respect to the surface area of the plate. On rectangular plates, 155 ul of the bacterial culture was mixed with 45 ul of phage lysate at a dilution determined to yield at least 10 plaques. On circular plates, 620 uL of the bacterial culture was mixed with 45 ul of phage lysate. For a single T2/Eco4 condition on a circular plate, we used 135 ul of phage lysate for both the experimental and control conditions to achieve the at least 10 plaques. After incubating at room temperature without shaking for 5 min, the mixture was added to 2 ml melted MMB agar and poured onto MMB agar plates. The plates were dried for 20 min at room temperature.

Plates were incubated overnight at 37°C. Plaque forming units were blindly counted and compared between the defense and control strains. To achieve at lest 10 individual countable plaques, the cell culture volume added was optimized to get a full bacterial lawn for each strain.

## RESULTS

### Endogenous RNase H1 determines retron RT-DNA length

Given that endogenous RNase H1 is required to remove RNA, resulting in single stranded retron RT-DNA, we aimed to test whether RNase H1 affects RT-DNA synthesis across a panel of retrons. For this work, we used a B-strain *E. coli* (BL21-AI) that harbors an endogenous retron (retron-Eco1). To create a clean background for the expression of multiple retrons, we eliminated the endogenous retron operon, and then additionally removed endogenous RNase H1 (rnhA), to yield the two primary strains used in this work: BL21*^Δ^^Eco1^* and BL21*^Δ^^Eco1;^^Δ^^rnhA^*. To understand the generalizability of RNase H1′s role in RT-DNA synthesis, we analyzed the effect of RNase H1 on RT-DNA synthesis in six retrons: Retron-Eco1 (Ec86), Retron-Eco2 (Ec67), Retron-Eco4 (Ec83), Retron-Eco6 (Ec48), Retron-Eco9 and Retron-Sen2 (St85). These six retrons span four RT Clades, from four different retron subfamilies, with four different types of predicted accessory domains (2,5). Clade, subfamily, and accessory type for each retron are provided in Supplementary Table S5.

Each retron operon, comprising of the retron-RT, retron-RNA, and accessory protein(s), was cloned into T7-inducible expression vectors and transformed into BL21*^Δ^^Eco1^* and BL21*^Δ^^Eco1;^^Δ^^rnhA^* cells. Retron operons were overexpressed and RT-DNA was isolated and visualized on a TBE-urea gel. (Figure 1B). In the presence of RNase H1, all retrons generate RT-DNA products of approximately the expected length based on previous literature - Eco1 (reported as 86 bases) (33), Eco2 (reported as 67 bases) (34), Eco4 (reported as 79 bases) (35), Eco6 (reported as 48 bases) (36,37), Eco9 (reported between 50 and 100 bases) (1), and Sen2 (reported between 50 and 100 bases) (38,39). Five of the six retrons tested displayed dramatically different RT-DNA profiles in the absence vs. presence of RNase H1. Eco4 RT-DNA is nearly eliminated in the absence of RNase H1. In contrast, Eco2 appears to be unaffected by RNase H1 (Figure 1B).

PAGE gel analyses have been the standard in the retron field, and provide an estimate of the size of the most prevalent polynucleotides in the population. However, PAGE gels are unable to quantify the lengths of molecules in a heterogenous population and provide no sequence information. Therefore, we applied multiplexed Illumina sequencing to the retron RT-DNA to provide a more quantitative and precise view of RT-DNA production across conditions.

We isolated the RT-DNA produced *in vivo* from each retron and processed them for sequencing using an unbiased custom sequencing method (20). Briefly, RT-DNA samples were processed with DBR1 to remove the 2′-5′ linkage present in some RT-DNA samples. Each sample was extended on the 3′ end with adenine bases using a template-independent polymerase (TdT), after which an adapter-containing primer was used to generate the complementary strand via Klenow extension, and finally a separate adapter was ligated to the other end of the resulting dsDNA. The resulting fragments were indexed and sequenced on an Illumina MiSeq instrument.

For a quantitative analysis, it is critical that all molecules are equally prepared for sequencing with no bias based on RT-DNA sequence. TdT has been shown to incorporate different nucleotides at different rates (40), and, although we were only using a single nucleotide for extension, we were concerned that the identity of the terminal RT-DNA base might skew our quantitative sequencing results. To test this potential bias, we synthesized and sequenced three sets of four oligonucleotides with each of the four possible terminal bases, mixed at an equal concentration. When adenine was used as the extension base (as described above), we found no bias in the ratio of terminal bases. In contrast, if C was used as the extension base, oligos terminating in C were overrepresented (Supplementary Figure S1).

For data analysis, lengths of sequences containing at least 10 bases specific to the retron of interest were counted. A binned histogram with the length of each retron RT-DNA in the presence and absence of RNase H1 is shown in Figure 1C–F. Plotting the length distribution of the sequences confirmed that all retrons produced a nearly homogenous population of RT-DNA in the presence of RNase H1 (Figure 1C–H). In the absence RNase H1, the distribution of the RT-DNA’s length was much broader for retrons Eco1, Eco6, Eco9 and Sen2. Eco2 showed no change in length, consistent with PAGE gel result, and Eco4 produced so little RT-DNA in the absence of RNase H1 that it could not be reliably prepared for sequencing. Based on these analyses, endogenous RNase H1 consistently affects the length distribution of RT-DNA molecules.

### RNase H1 determines sites of termination in a retron dependent manner

**Figure 2.**
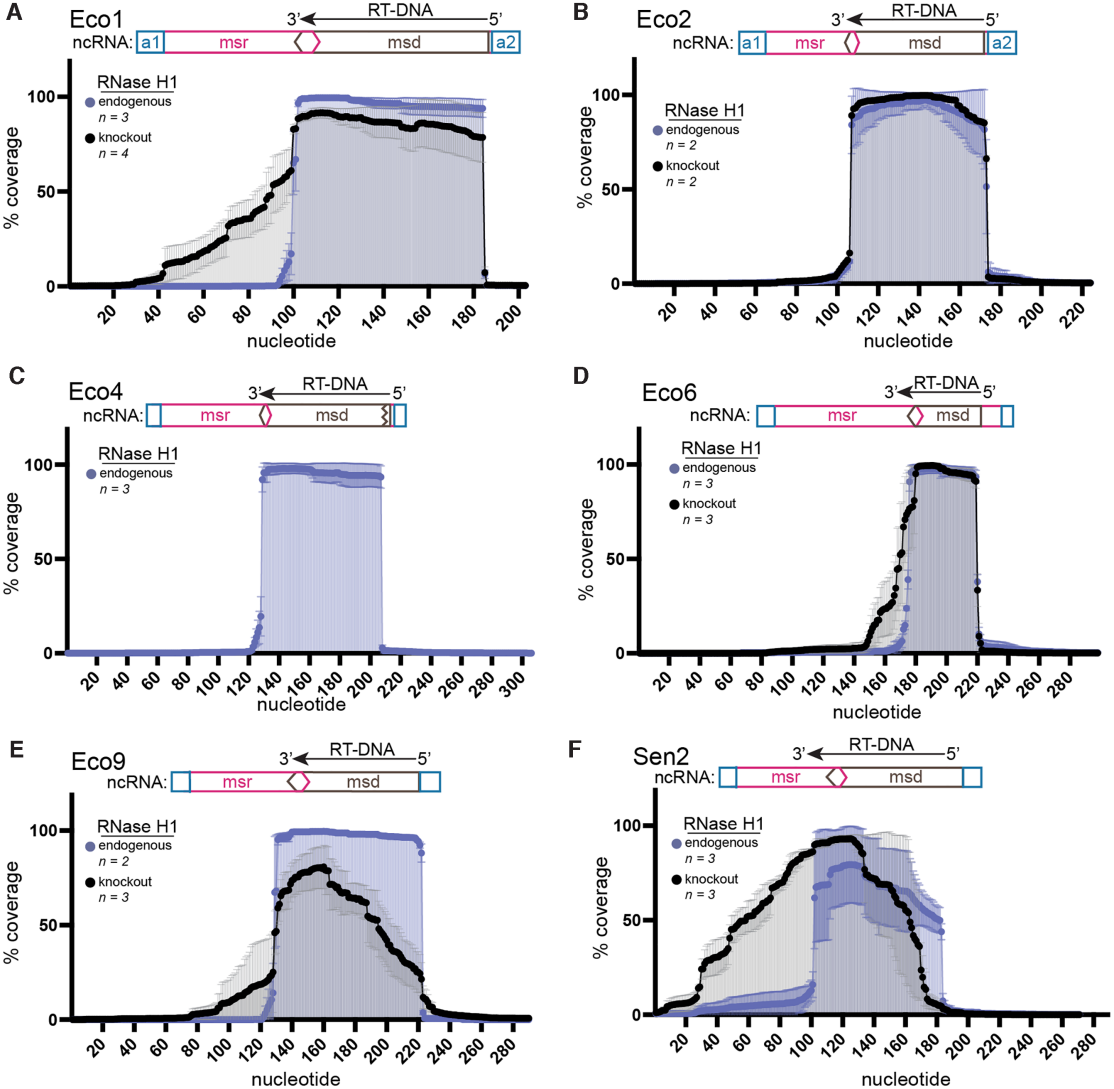
RNase H1 determines sites of termination in a retron dependent manner. (**A–F**) Reads containing retron RT-DNA sequences were assembled onto the ncRNA sequence and flanking regions. Samples with endogenous RNase H1 are shown in blue and RNase H1 knockout experiments are shown in black (±SD). A schematic of the proposed ncRNA is positioned above the alignment with the inverted repeat a1/a2 region in blue, the msr portion in pink and the msd in brown. Retron name is indicated for each panel at the top left.

Visualization by PAGE gel and length analysis of multiplexed sequencing data show that, in the absence of RNase H1, RT-DNA length is highly heterogeneous for four retrons. This heterogeneity suggests that in the absence of RNase H1, the retron RT may load onto or fall off the template at different places, skip and/or duplicate various portions of the template, or continue beyond the normal stop site. To distinguish between these possibilities, we aligned the trimmed reads to a reference sequence containing the retron ncRNA. The analysis showed that in the presence of RNase H1, most retrons produced RT-DNA with little variation in the site of initiation and termination (Figure 2). Schematics show canonical regions of the retron ncRNA, including the flanking a1/a2 inverted repeats, non-reverse transcribed region (msr), and reverse transcribed region (msd) for context. In the absence of RNase H1, RT-DNA was created without duplications or deletions, but the typical tightly defined termination point at the 3′ end – with the majority of molecules terminating within a span of ≤ 3 bases – was lost for retrons Eco1, Eco6, Eco9, and Sen2 (Figure 2A, D–F). We additionally found greater variability during initiation at the 5′ end for Eco9 and Sen2 (Figure 2A, D–F). The variability on the 5′ side of the DNA could be due to changes in the ability of the RT to initiate reverse transcription properly. These results indicate that the changes in RT-DNA length observed in the absence of RNase H1 are due to differences in the 5′ end and 3′ termination site.

We additionally compared our RT-DNA sequencing results to the RT-DNA of these retrons from their initial characterization in previous literature, except for Eco9, which does not have a previously described RT-DNA sequence (Supplementary Figure S2A–F). The termination points were as described except for Eco1 which produced RT-DNA that was one base shorter than was previously described, resulting in an RT-DNA that is 85 bases rather than 86 (33) (Figure 2A; Supplementary Figure S2A). On the 3′ end, in contrast to what was previously reported, we found that Eco6 was shorter by three bases (36) (Supplementary Figure S2D). Eco4 and Eco9 produce unbranched RT-DNA, in which the 2'-5' linkage is removed, and our data was consistent with the post-processing cleavage point as previously described (1,35). We assume the differences that we report in RT-DNA compared with the previous literature represent a technical refinement gained by sequencing thousands of RT-DNA molecules individually instead of using bulk assays, and not a biological difference.

### *In vitro* validation of RNase H1 as a requirement for correct termination

It has been proposed that retrons form large complexes (200–600 kDa) composed of both protein and RNA in their bacterial hosts (41,42). Therefore, RNase H1 could be directly responsible for the change in RT-DNA length or be indirectly modifying the RNP complex by recruiting an additional co-factor. To distinguish between these two hypothesizes, we optimized a minimal *in vitro* reverse transcription assay from purified components.

**Figure 3.**
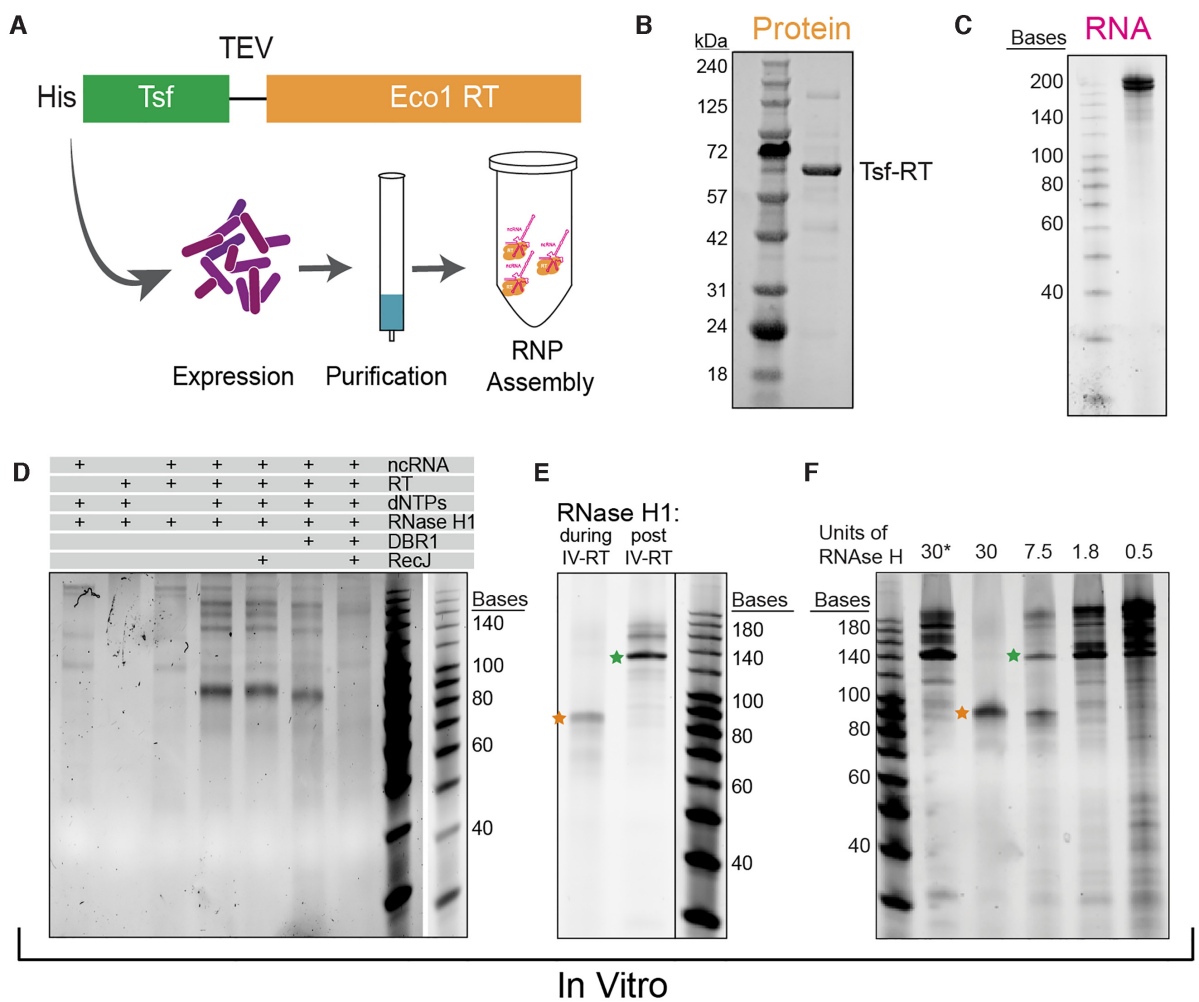
*In vitro* validation of RNase H1 as a requirement for correct termination. (**A**) Schematic showing the purification of Eco1 RT (orange) with a His/Tsf tag on the N-terminus with a TEV linker. (**B**) SDS-PAGE gel of purified Retron-RT. (**C**) TBE-urea gel of *in vitro* transcribed RT-RNA. (**D**) *In vitro* production of RT-DNA. Components of the reaction are shown above. RT-DNA of ∼85 bases is produced in the presence of Eco1 ncRNA, RT, dNTPs and RNase H1. This RT-DNA is protected at the 5′ end against RecJ, debranched by DBR1, and degraded by RecJ after debranching. RNase A/T1 was added to all reactions before analysis on a TBE–urea gel. (**E**) Gel showing RT-DNA production when RNase H1 is present during the reaction vs. when it is added after the reaction has been quenched. Orange star represents the canonical length of Eco1 RT-DNA and the green star represents the RT-DNA product that does not terminate correctly. (**F**) RT-DNA production with a titration of RNase H1 present during the reaction. Asterisk indicates that RNase H1 was added after the reaction.

First, we expressed a 6X histidine (His)-tagged Eco1 retron RT for purification, with a Tsf solubility domain linking the His-tag to the N-terminus of the RT (Figure 3A). Retron Eco1 RT was expressed for 18 h at 18°C and isolated using a nickel affinity resin followed by a size exclusion column (Figure 3B). Addition of the Tsf domain does not affect RT-DNA production *in vivo* (Supplementary Figure S3A), therefore it was not removed. Next, Eco1 Retron-ncRNA was *in vitro* transcribed using T7 polymerase (Figure 3C). To confirm that the purified RT was functional and producing 2′-5′ linked RT-DNA, we performed an *in vitro* reverse transcription assay, looking for the presence of RT-DNA by PAGE analysis. In this experiment, the components were added to the reaction systematically and the reaction was allowed to proceed for 1 h at 37°C. After 1 h, the sample was treated with RNase A/T to quench the reaction and degrade RNA. DNA products were visualized on a TBE-urea gel. We observe *in vitro* production of RT-DNA at the expected size in the presence of Eco1 ncRNA, RT, dNTPs and RNase H1 (Figure 3D).

*In vivo*, Eco1 RT-DNA is covalently linked to the RNA via a 2′-5′ linkage (33). To confirm that purified RT also produced 2′-5′ linked RT-DNA, the *in vitro* reaction was treated with RecJ, a 5′→3′ ssDNA exonuclease. In this condition, the RT-DNA persists, indicating the RT-DNA produced is protected at its 5′ end (Figure 3D). By contrast, incubation with DBR1, an enzyme that cleaves 2′-5′ linkages, led to a slight shift in size, consistent with the removal of the linkage and the four bases of RNA that remain after RNase A/T1 treatment (Figure 3D). In the presence of both DBR1 and RecJ, the band was degraded due to the de-protection of the 5′ end by DBR1 (Figure 3D). Together, these experiments confirm that the purified Eco1 RT protein produces 2′-5′ linked retron-derived RT-DNA of the correct length.

To test the requirement of RNase H1 for RT-DNA production *in vitro*, we performed a reverse transcriptase reaction in the presence and absence of RNase H1. We post-treated both reactions with RNase H1 to avoid complicated banding patterns due to single vs double stranded products and ran each reaction on a TBE Urea gel. Like the *in vivo* system, omitting RNase H1 during reverse transcription led to the production of a longer RT-DNA (Figure 3E). By titrating the amount of RNase H1 present during the reaction, we could modify the ratio of short to long RT-DNA (Figure 3F). This experiment demonstrates that in a minimal *in vitro* system, RNase H1 alone is capable of modulating the length of Eco1 RT-DNA. Interestingly the band found at ∼140 is also sometimes present in in vivo RT-DNA production experiments (Supplementary Figure S3B), demonstrating that in over-expression conditions *in vivo*, RT-DNA may saturate the presence of endogenous RNase H1 resulting in these higher order products.

### Secondary structure in Eco1 ncRNA is not responsible for termination

The loss of RNase H1 resulted in reverse transcription past the canonical termination point in four retrons tested: Eco1, Eco6, Eco9 and Sen2. One possible explanation is that RNase H1 could physically block the progress of the RT, preventing additional reverse transcription. To test the steric blocking hypothesis, a catalytically dead RNase H1 (RNase H1^D10N^) was constructed by mutating one of the conserved catalytic residues (D10N) (43). We replaced the endogenous RNase H1 in the knockout background (BL21*^Δ^^Eco1;^^Δ^^rnhA^*) with either RNase H1^D10N^ or RNase H1^wt^ driven by a constitutive promoter and the Eco1 RT driven by a promoter driving over-expression. The presence of the RNase H1^wt^ rescued the production of Eco1 RT-DNA at a single length when analyzed by PAGE gel, while RNase H1^D10N^ did not (Supplementary Figure S4A). While we cannot rule out the possibility that the D10N mutation caused a slight protein structural change resulting in a loss of binding to the RT, we believe that the more likely explanation for this result is that removal of the RNA component of the DNA:RNA hybrid formed upon reverse transcription is necessary for correct termination.

**Figure 4.**
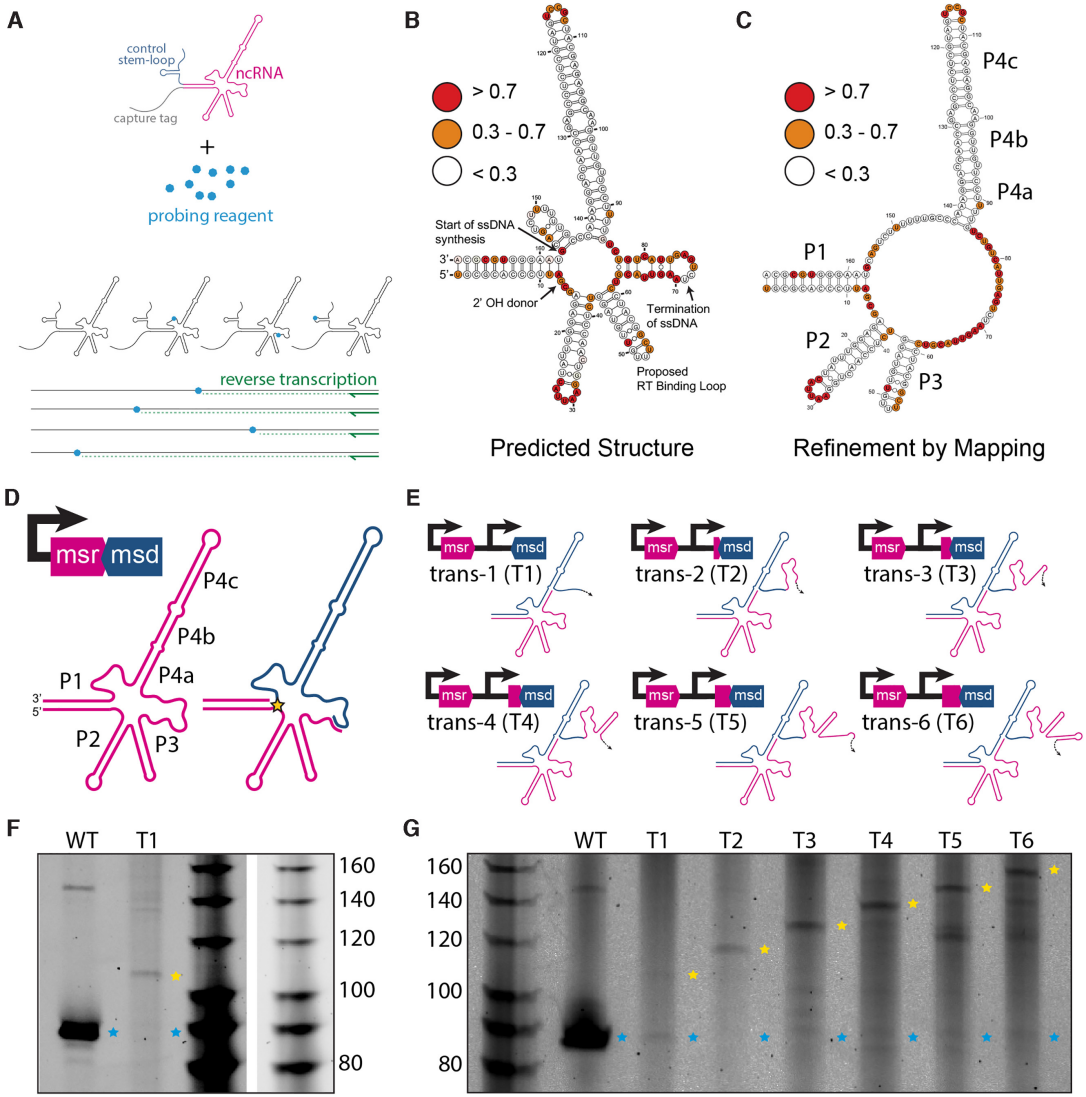
Secondary structure in Eco1 ncRNA is not responsible for termination. (**A**) Schematic of chemical mapping experiment. (**B**) Chemical mapping binned reactivity on the *in silico* predicted secondary structure. Red indicates a reactivity value above 0.7, orange 0.3–0.7 and white below 0.3. The base that donates the -OH group to form the 2′-5′ linkage, the start of the template used for ssDNA synthesis, the canonical termination point and the proposed RT binding loop are indicated on the secondary structures with arrows. (**C**) Chemical mapping binned reactivity on a refined structure. (**D**) Eco1 ncRNA with verified stems labeled. Pink indicates the msr region and blue the msd region. The star indicates the 2′-5′ linkage. (**E**) Schematic of trans ncRNA architectures and operon architectures. Colors of schematics match ‘D’. (**F**) TBE-Urea gel of WT ncRNA and ncRNA trans-1 (T1). Blue stars show WT Retron-RNA size and yellow stars show an RT-DNA that does not terminate at the correct site. (**G**) TBE-urea gel of constructs T1-T6 using the same star nomenclature as 'F'.

Another model for termination has been proposed, which suggests that the retron secondary structure is responsible for RT termination (44). When Eco1 ncRNA is folded *in silico* using RNAstructure (45), the termination site lands inside a loop on a short predicted hairpin (Supplementary Figure S4B). To test whether this hairpin exists, we evaluated the secondary structure using chemical mapping (Figure 4A). In this experiment, *in vitro* transcribed retron ncRNA is exposed to 1-Methyl-7-nitroisatoic anhydride (1M7). Nucleotides that are not paired have more flexible phosphate backbones and therefore are reactive to 1M7, resulting in the addition of a bulky side group at the 2′hydroxyl of the phosphate backbone (46). Conversely, nucleotides that are base-paired have backbones that are more rigid and therefore are not reactive to 1M7. After the 1M7 reaction, a commercial reverse transcriptase (Superscript III) and primer are added. Nucleotides that have been modified by 1M7 prevent Superscript III from reverse transcribing beyond the modified nucleotide. Analyzing the length of reverse-transcribed products yields a reactivity profile highlighting nucleotides that are not base paired to evaluate secondary structure.

To perform this experiment, Eco1 ncRNA was modified to include a binding site for the Superscript III reverse transcriptase and a hairpin for normalization of reactivity across experimental replicates (Supplementary Figure S4C) (24). Addition of these sites did not affect Eco1 function in an *in vitro* functional assay (Supplementary Figure S4D). Eco1 ncRNA chemical mapping largely supports the *in silico* secondary structure prediction (Figure 4B) with a lack of reactivity in four major stem regions (counter-clockwise from left: P1, P2, P3, P4a, P4b, P4c) and reactivity in the loops of P2, P3 and P4c (see Figure 4D for labeling). Conversely, there was a very high level of reactivity on the *in silico* predicted hairpin where the RT-DNA termination occurs between P3 and P4, suggesting that this hairpin is not stably formed (Figure 4B). Surprisingly, there is a lack of reactivity in the bulge regions separating P4a/P4b and P4b/P4c and a more complicated reactivity pattern is observed between P1 and P4, where the RT-DNA synthesis begins. Based on these data, the secondary structure can be more accurately represented without the predicted hairpins between P1/P4 and P3/P4 (Figure 4C, D).

The chemical mapping results indicate it is unlikely that a stem structure exists at the Eco1 termination point. However, it is possible that another secondary structure downstream is responsible for termination. To test this hypothesis, we constructed a vector where the portion of the ncRNA that is not reverse transcribed (msr) is separated from the reverse transcribed region of the ncRNA (msd) and these two pieces are expressed from two different promoters with the intention of creating an msr and an msd in trans, rather than their endogenous cis architecture. (Figure 4E). While we cannot rule out some read-through transcription that would create one transcript, the msr and msd on that transcript would still be separated by more than 200 bases, including a promoter, which we would consider to mimic the intended trans architecture. Remarkably, ncRNA produced from these two promoters is still functional for RT-DNA production, albeit at a lower level than the cis construct (Figure 4E, F). However, rather than terminating at the canonical point, reverse transcription continues to the 5′ end of the template RNA (Figure 4E, F).

These results provide a different way to disrupt the RT-DNA termination even in the presence of RNase H1, indicating that the termination requires either a downstream secondary structure or the overall tertiary structure of the endogenous ncRNA. To distinguish between these possibilities, we created a construct where an increasing number of bases from the msr sequence of the ncRNA were added back to the msd (template) in the trans architecture (Figure 4E). If a secondary structure found downstream on the ncRNA is important for termination, correct termination should be restored when the sequence encoding this structure is included. However, these constructs never regained the ability to terminate correctly, and only led to increasingly long RT-DNAs (Figure 4E). Based on these experiments, we propose a model in which the tertiary structure of Eco1 ncRNA, rather than a secondary structure, determines the termination point and that removal of template RNA by RNase H1 is required for that tertiary structure to form.

### Absence of RNase H1 affects accessory protein toxin neutralization

Bobonis *et al.* (1) recently reported that the accessory proteins often present in retron operons can act as toxins that are neutralized in the presence of the retron's RT and RT-DNA (1,2). Retron accessory proteins were categorized based on their domains' predicted function (5). The accessory proteins of Eco1, Eco9 and Sen2 are predicted to have N-terminal nucleoside deoxyribosyltransferase-like (NDT) and C-terminal DNA binding domains (5). Eco2 is fused to its accessory protein which is predicted to be a topoisomerase-primase domain (5). Eco4′s accessory protein is predicted to have ATPase and Walker A and B motifs (5). Eco6 has an accessory protein that is predicted to be an integral membrane protein with two transmembrane helices (5).

**Figure 5.**
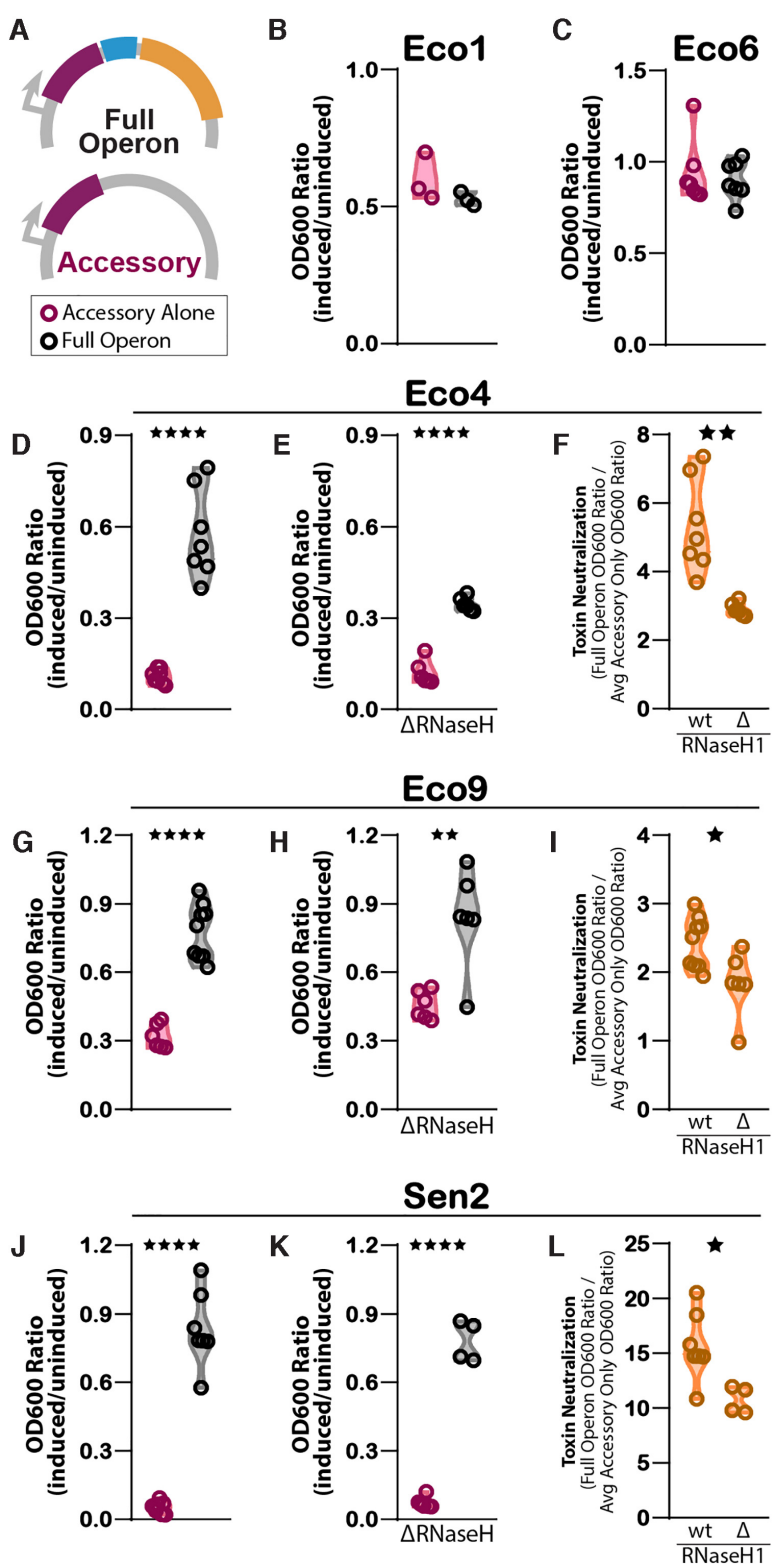
Absence of RNase H1 affects neutralization of accessory toxin. (**A**) Schematic of full Eco1 operon (accessory in maroon, ncRNA in blue, and RT in yellow) and accessory only constructs. (**B**) Impact of expression of Eco1 full operon (black) and accessory only (maroon) on cell growth in BL21*^Δ^^Eco1^*strain. Growth is measured by OD_600_ and normalized to the uninduced condition for each construct. Individual points are biological replicates. (C) Impact of expression of full Eco6 operon and accessory-only on cell growth in BL21*^Δ^^Eco1^*strain. (**D**) Impact of Eco4 full operon and accessory only expression on cell growth in BL21*^Δ^^Eco1^*strain. (**E**) Impact of full Eco4 operon and accessory-only expression on cell growth in BL21*^Δ^^Eco1;^^Δ^^rnhA^*strain. (**F**) Magnitude of toxin neutralization (full operon OD_600_ ratio (induced/uninduced) over accessory only OD_600_ ratio (induced/uninduced) as a function of strain genotype. (**G**) As in (D), but for Eco9. (**H**) As in (E), but for Eco9. (**I**) As in (F), but for Eco9. (**J**) As in (D), but for Sen2. (**K**) As in (E), but for Sen2. (**L**) As in (F), but for Sen2. Statistical details in Supplementary Table S1.

We found evidence that the RT and the accessory protein in retron Eco1 interact, either directly or as part of a complex, based on mass spectroscopy analysis of FLAG-tagged, overexpressed Eco1-RT, which co-immunoprecipitates with endogenous accessory protein, similar to what is observed in Sen2 (1) (Supplementary Figure S5). However, whether the RT-DNA interacts with the accessory protein as part of this neutralization is unknown. Given that removal of RNase H1 modifies the RT-DNA produced, we wondered whether it would affect toxin neutralization. To test this, we generated inducible constructs that contained either the retron accessory protein alone or the full retron operon for three different families of RT-accessory protein pairs, represented by Eco1, Eco4, Eco6, and Eco9/Sen2. Each construct was then transformed into either BL21*^Δ^^Eco1^* or BL21*^Δ^^Eco1;^^Δ^^rnhA^*. Cells were grown in either the presence or absence of inducers over 24 h at room temperature, and the effect of each construct on cell growth was assessed by measuring the optical density (Figure 5A).

Surprisingly, the Eco1 and Eco6 accessory proteins did not appear to be toxic, as the full operon and accessory protein only strains grew to the same relative density (Figure 5B, C). In contrast, the Eco4 accessory protein had a very strong toxic effect on cell growth (Figure 5D). The growth defect caused by the accessory protein was largely neutralized by expressing the full operon, similar to what has been previously reported for Eco9 and Sen2 (1). When the Eco4 accessory protein only constructs were expressed in BL21*^Δ^^Eco1;^^Δ^^rnhA^*, a toxic affect was also observed and, again, this toxic effect was mitigated by expressing the full operon (Figure 5E). We next analyzed the degree of toxin neutralization by the full operon in each strain, defined as the full operon OD_600_ ratio (induced/uninduced) over the accessory alone OD_600_ ratio (induced/uninduced). We found that the degree of toxin neutralization in the *BL21^Δ^^Eco1;^^Δ^^rnhA^* strain was lower than in the BL21*^Δ^^Eco1^* strain (Figure 5F). A similar effect was observed for Eco9 and Sen2. The accessory protein constructs exhibited a toxic effect on cell growth that is largely neutralized when the full operon is expressed (Figure 5G, J), and both the toxic effect and neutralization persist in the BL21*^Δ^^Eco1;^^Δ^^rnhA^*strain (Figure 5H, K). However, the degree of toxin neutralization is reduced in the BL21*^Δ^^Eco1;^^Δ^^rnhA^*strain compared to the BL21*^Δ^^Eco1^* strain (Figure 5I, L). We conclude that RNase H1 is required for maximal accessory neutralization in retrons where the accessory has a toxic phenotype.

### Absence of RNase H1 inhibits retron-based phage defense

**Figure 6.**
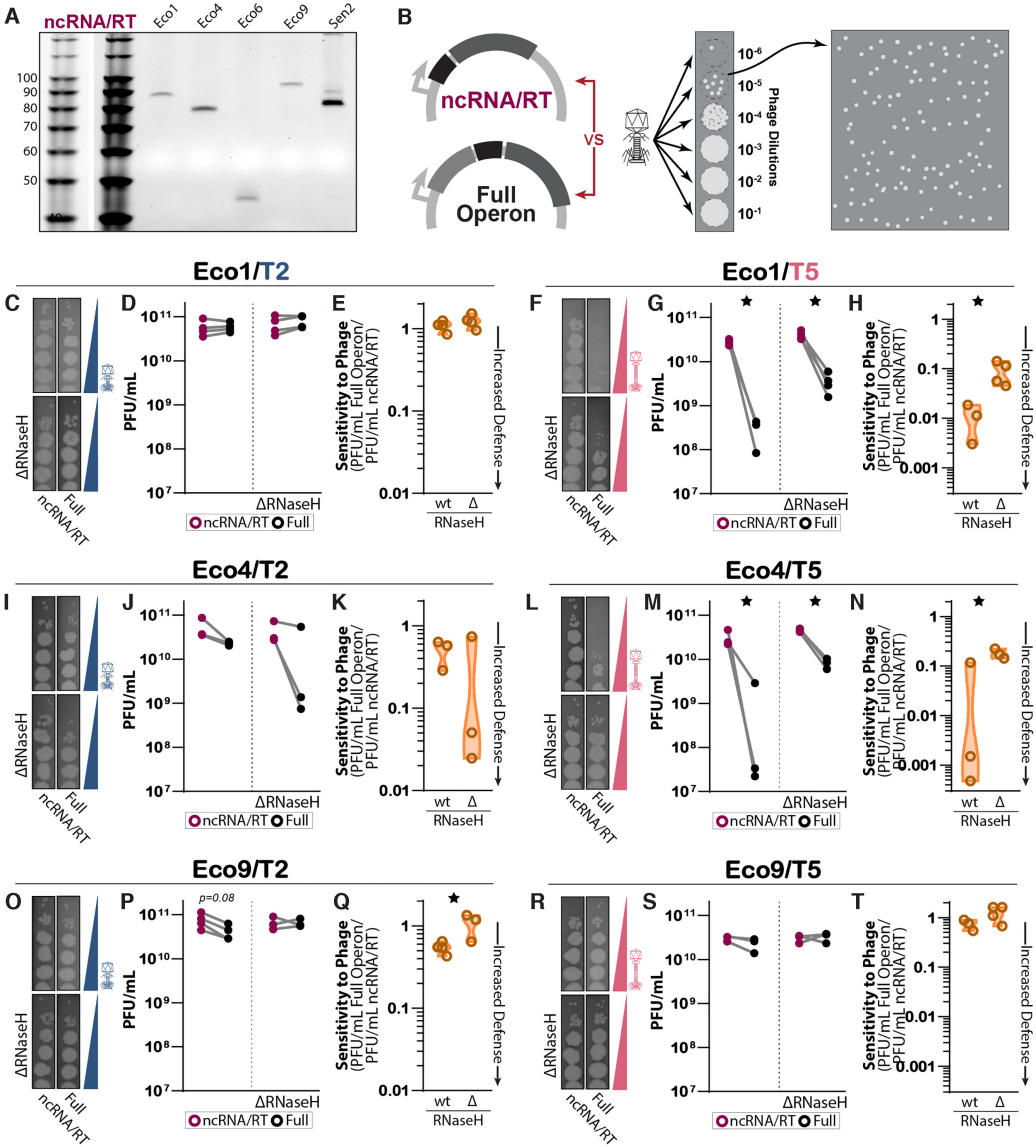
The absence of RNase H1 inhibits retron-based phage defense. (**A**) TBE–urea gel showing production of RT-DNA in the absence of the accessory protein. (**B**) Schematic of full operon and ncRNA/RT constructs used in plaque assays and quantification of plaque forming units (PFU). Phage lysates were titrated onto plates of each strain/construct pair to find a concentration that yields distinct plaques. That concentration was then tested over a full plate where plaques were counted. The count was converted into PFU per mL of phage lysate. (**C**) Titration of phage T2 on strains containing Eco1 constructs with and without RNase H1. (**D**) PFU/mL of T2 on strains containing Eco1 constructs with and without RNase H1. Biological replicates of the full operon (black) and ncRNA/RT (magenta) constructs that were performed in parallel are connected with a gray line. (**E**) The sensitivity of each strain to T2, as expressed by the ratio of plaque-forming units on strains with the full operon versus ncRNA/RT constructs, in the presence or absence of RNase H1. A sensitivity value of 1 indicates no retron-based defense, while a value of 0 indicates total retron-based defense. Generally, as retron-based defense strengthens (approaches 0), the strain's sensitivity to phage decreases. For Eco1, there is effectively no defense against T2 and therefore no difference between strains. (**F**) Titration of T5 on strains containing Eco1 constructs with and without RNase H1. (**G**) PFU/mL of T5 on strains containing Eco1 constructs with and without RNase H1. (**H**) Sensitivity values indicate a significant difference in Eco1 defense against T5 with and without RNase H1. (**I**) Titration of T2 on strains containing Eco4 constructs with and without RNase H1. (**J**) PFU/mL of T2 on strains containing Eco4 constructs with and without RNase H1. (**K**) Sensitivity values indicate that there is no significant difference in defense between strains. (**L**) Titration of phage T5 on strains containing Eco4 constructs with and without RNase H1. (M) PFU/mL of T5 on strains containing Eco4 constructs with and without RNase H1. (**N**) Sensitivity values indicate a significant difference in Eco4 defense against T5 with and without RNase H1. (**O**) Titration of T2 on strains containing Eco9 constructs with and without RNase H1. (**P**) PFU/mL of T2 on strains containing Eco9 constructs with and without RNase H1. (**Q**) Sensitivity values indicate a significant difference in Eco9 defense against T2 with and without RNase H1. (**R**) Titration of T5 on strains containing Eco9 constructs with and without RNase H1. (**S**) PFU/mL of T5 on strains containing Eco9 constructs with and without RNase H1. (**T**) For Eco9, there is effectively no defense against T5 and therefore no difference between strains.

Retrons have been shown to confer defense against phages, which requires all three components: RT, RT-DNA and accessory protein. To probe the effect of RNase H1 on retron-based phage defense, we created two constructs: a full retron operon and an ncRNA/RT-only construct for Eco1, Eco4, and Eco9. We then tested these constructs against two phages, T2 and T5. We validated that RT-DNA of the same size was still produced in the absence of the accessory protein (Figure 6A). Induced bacterial cultures expressing each individual construct were plated to create a full lawn of bacteria. A titration of phage was then spotted onto the plate and bacteria were allowed to grow overnight. Plaque formation across this titration yielded an initial qualitative assessment of the overall effect that each construct had on phage growth and enabled us to identify the appropriate phage concentration for more quantitative analysis (Figure 6B).

For quantitative analysis, bacterial cultures were plated in the presence of phage at a concentration that allowed at least ten distinct plaques to form, and plaques were counted blind to condition. Eco1 in both the BL21*^Δ^^Eco1^* or BL21*^Δ^^Eco1;^^Δ^^rnhA^*conditions displayed no difference in phage defense between the ncRNA/RT and full operon constructs against T2 phage (Figure 6C–E). However, Eco1 did display defense against T5, consistent with previous reports, with far fewer plaques when the full operon was expressed than the ncRNA/RT was expressed alone (Figure 6F–H) (2). This defense phenotype was reduced in the BL21*^Δ^^Eco1;^^Δ^^rnhA^*strain as compared with the BL21*^Δ^^Eco1^* strain, indicating a role for RNase H1 in retron phage defense.

We found that the full Eco4 operon yielded significant defense against T5 versus the ncRNA/RT alone, though prior work suggests that Eco4 defends against T2, but not T5 (2). For Eco4, like Eco1, the magnitude of defense against T5 was lower in the BL21*^Δ^^Eco1;^^Δ^^rnhA^*strain than in the BL21*^Δ^^Eco1^* strain (Figure 6I–N). A phage phenotype for Eco9 has not been previously established. Our data demonstrate that the full Eco9 operon defends modestly against T2 (Figure 6O–T) and that this defense is also significantly diminished in the BL21*^Δ^^Eco1;^^Δ^^rnhA^* strain compared to the BL21*^Δ^^Eco1^*strain. Overall, these results point to a general role for RNase H1 in retron-based phage defense across retron subtypes and phages.

## DISCUSSION

In this study, we demonstrate that RNase H1 plays a fundamental role in the mechanism of retron reverse transcription and phage defense. RNase H1 dictates RT-DNA length, with other subtle effects related to RT-DNA production depending on the specific retron. Additionally, we characterized RT-DNA from six retrons using multiplexed sequencing to provide a quantitative characterization of the RT-DNA population in the presence and absence of RNase H1. The most severely affected retron is Eco4, where RT-DNA is nearly eliminated in the absence of RNase H1. Eco1, Eco6, Eco9 and Sen2 all generate RT-DNA products of non-canonical lengths, reminiscent of early experiments with Eco5 (14,15), whereas Eco2 is unaffected by RNase H1, similar to what was previously observed for Eco7 (14,15).

We advance the understanding of retron termination, showing that termination is not mediated by a steric effect of RNase H1, or by secondary structure of the ncRNA. Instead, we propose that termination is mediated by tertiary structure that forms upon removal of the template RNA by RNase H1. Finally, we show that not all retron accessory proteins are toxic, but when they are, RNase H1 affects the ability of retron RT-DNA to neutralize the toxic effect. Regardless of whether the accessory protein is toxic or not, the activity of RNase H1 affects ability of the retron to defend bacterial populations against phages.

These experiments also yield insight into the specific mechanism of Eco1 termination. We performed experiments to test a previously proposed model in which secondary structure mediates termination (44). Our data does not support this model for Eco1 based on both direct evaluation of ncRNA secondary structure and the absence of termination in the circularly permutated constructs. We instead propose a model in which termination in Eco1 is achieved through the tertiary folding of the single-stranded RT-DNA/ncRNA template region. Supporting this hypothesis is the lack of reactivity in the bulge regions between P4a/b/c and the complex reactivity observed between region P1/4 and P3/4 in the Eco1 ncRNA. Additionally, retron termination was affected by both the absence of RNase H1 and the separation of the msr (primer) and msd (template) components of the ncRNA. These data are unified in a model where the single-stranded RT-DNA and remaining ncRNA scaffold folds into a tertiary structure that enforces precise termination.

While the proposed model of a toxic effect induced by the accessory protein for phage defense was supported by Eco4 and Eco9, no toxic phenotype was shown for accessory proteins of Eco1 and Eco6. This is not simply explained by accessory protein domain category, as Eco1 and Eco9 share the same accessory protein neutralization family (predicted NDT-DNA binding domain), whereas Eco6 is in an entirely different family (two transmembrane integral membrane protein). Interestingly, despite this lack of a toxic effect, Eco1 still exhibited a strong phage defense against T5. Taken together, this demonstrates that the current model for retron-based phage defense and the mechanism of action for accessory proteins, even among similar family members, is not universally applicable to all retrons.

It is interesting that the phage defense is reduced, but not entirely eliminated by the RNase H1 effect that substantially changes the RT-DNA. We interpret the effect of RNase H1 on toxicity to indicate that the fully single-stranded RT-DNA of the correct length is most effective in neutralizing the accessory protein. We assume that a similar mechanism underlies the effect on phage defense, although that mechanism must differ slightly in ways that we do not yet understand in systems like Eco1 where the accessory protein is not a direct toxin. The remaining phage defense may be due to a small fraction of RT-DNA molecules that are accurately produced even in the absence of RNase H1, or could indicate a diminished, but not eliminated role, for the inaccurate RT-DNA in phage defense.

## DATA AVAILABILITY

All data supporting the findings of this study are available within the article and its supplementary information, or will be made available from the authors upon request. Sequencing data associated with this study is available from NCBI with accession PRJNA784591.

Custom code to process or analyze data from this study will be made available on github at https://github.com/Shipman-Lab.

## Supplementary Material

gkac177_Supplemental_Files
