# Supplementary material for: Retron reverse transcriptase termination and phage defense are dependent on host RNase H1
Source: Nucleic Acids Res. 2022 Mar 16;50(6):3490–504. doi: 10.1093/nar/gkac177 (PMC8989520; doi:10.1093/nar/gkac177)
Supplement: gkac177_Supplemental_Files [file gkac177_supplemental_files.zip › Retron_RNaseH_Supplement_2022_02_21.pdf]

SUPPLEMENTARY DATA

Retron reverse transcriptase termination and phage defense are dependent on host RNase H1

Authors: Christina Palka[1,4], Chloe B. Fishman[1,4], Santi Bhattarai-Kline[1], Samuel A Myers[3], Seth L. Shipman[1,2]*

Affiliations:
[1]Gladstone Institute of Data Science and Biotechnology, San Francisco, CA, USA

[2]Department of Bioengineering and Therapeutic Sciences, University of California, San Francisco, CA, USA

[3]La Jolla Institute for Immunology, La Jolla, CA, USA

[4]These authors contributed equally

*Correspondence to: seth.shipman@gladstone.ucsf.edu

Figure S1

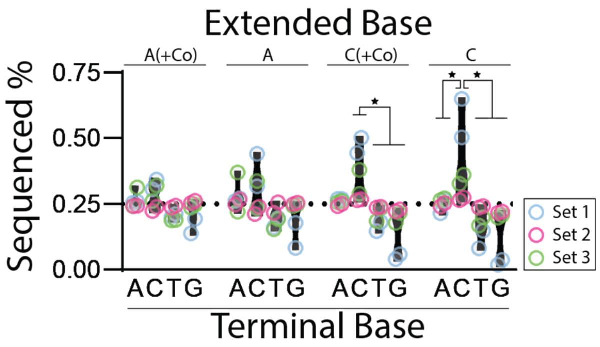

Figure S1: Sequenced ratios of oligos by terminal base, physically mixed at equal ratios, as a function of TdT extension base ± cobalt. Replicates of different oligo sets represented in different colored circles.

Figure S2

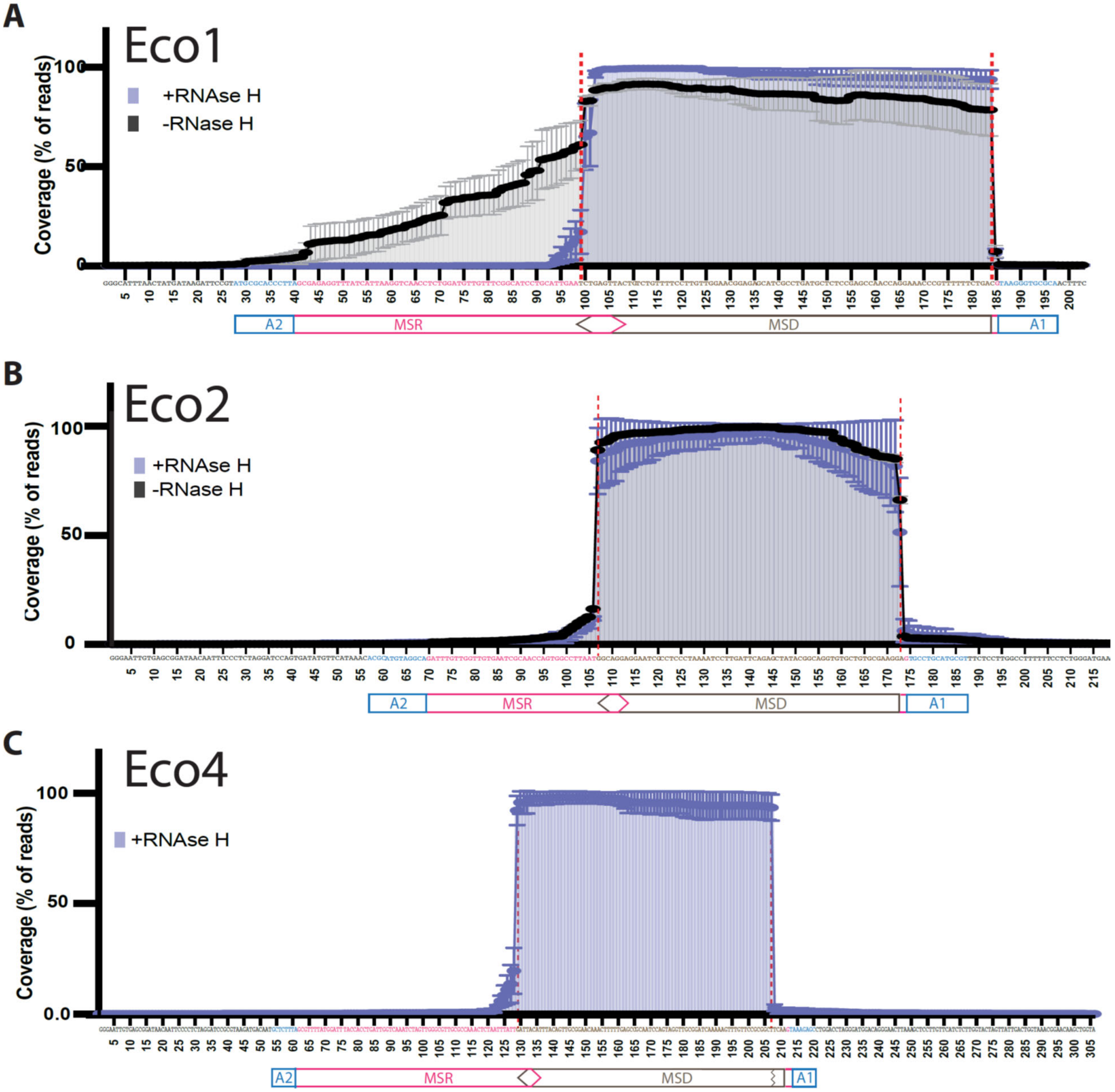
A
Eco1
Coverage (% of reads)
+RNAse H
-RNase H
A2
MSR
MSD
A1
B
Eco2
Coverage (% of reads)
+RNAse H
-RNase H
A2
MSR
MSD
A1
C
Eco4
Coverage (% of reads)
+RNAse H
A2
MSR
MSD
A1

Figure S2 - continued

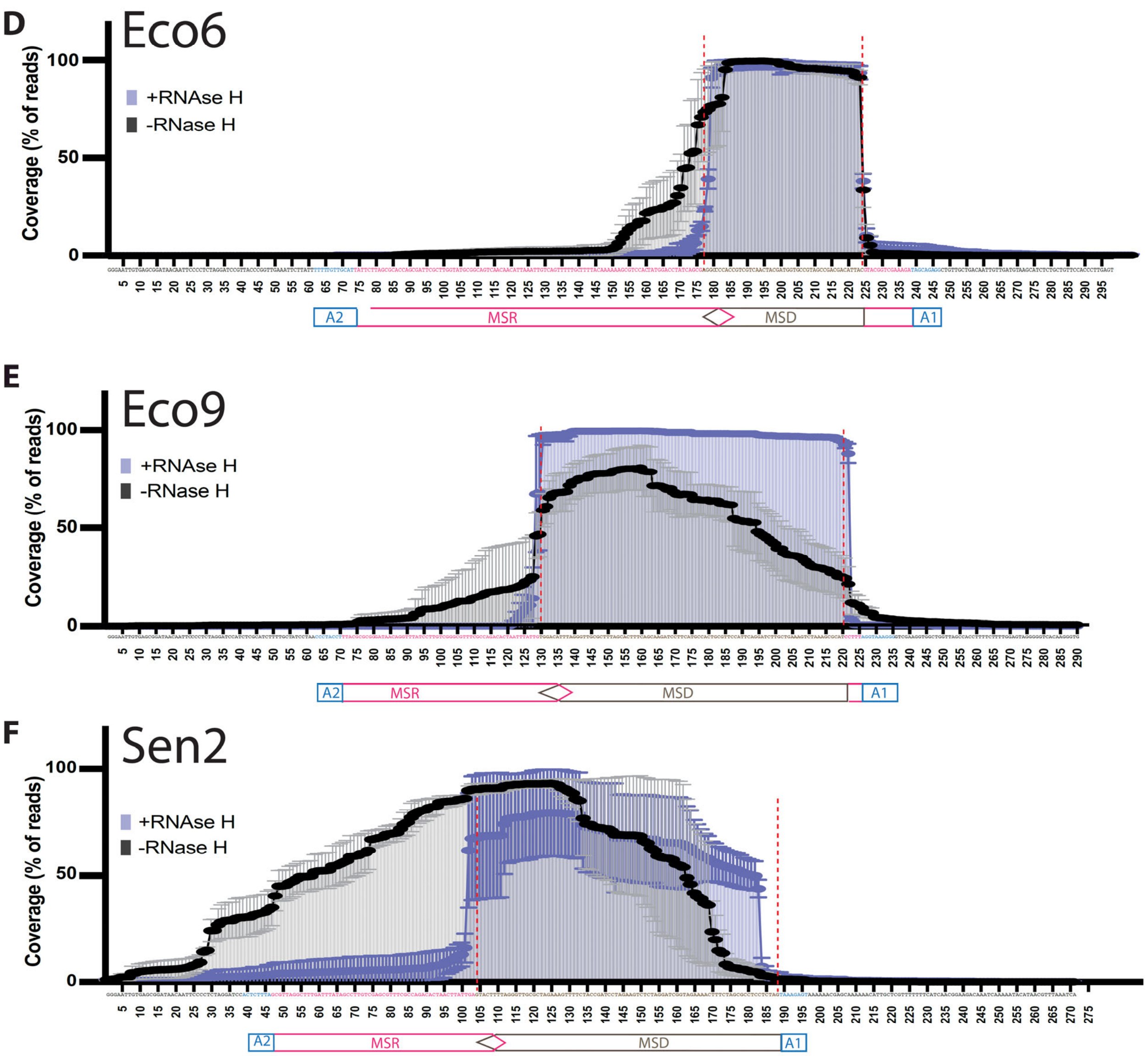

Figure S2. RT-DNA alignment for (A) Eco1, (B) Eco2, (C) Eco4, (D) Eco6, (E) Eco9, and (F) Sen2. Each retron RT-DNA containing sequence from Illumina sequencing reads was aligned against a sequence stretching from the T7 start site, through the ncRNA to the start of the RT start codon. Aligned profiles of reads derived from +RNase H1 conditions are in purple and -RNase H1 conditions in black. The nucleotide number and sequence are shown on the x-axis. A schematic of the ncRNA is shown below the graph with the region corresponding to the a1/a2 priming region shown in blue, the msr in pink, the msd in brown, and all other regions in black. The same color scheme is applied to the nucleotide sequence. Dotted red lines show the canonical retron RT-DNA initiation and termination sites.

Figure S3

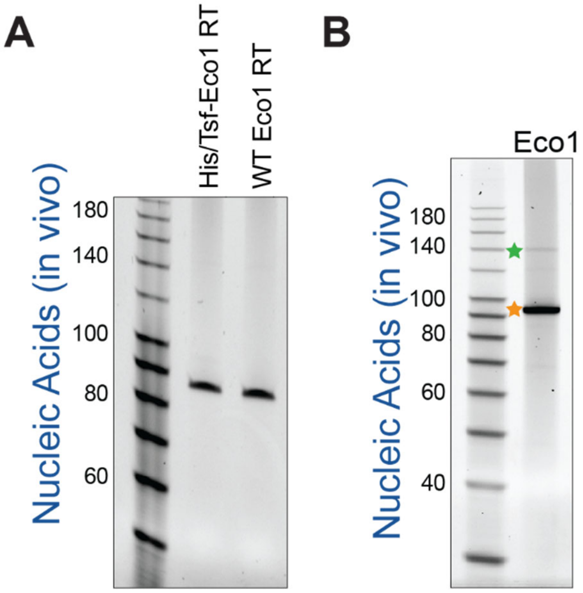

Figure S3. (A) WT or His/Tsf-tagged Eco1 was over-expressed and RT-DNA isolated and analyzed on a TBE-Urea gel. (B) WT Eco1 RT was overexpressed and RT-DNA was isolated. Canonical RT-DNA is denoted with an orange star and the higher order product sometimes observed in RT-DNA preps is detonated with a green star.

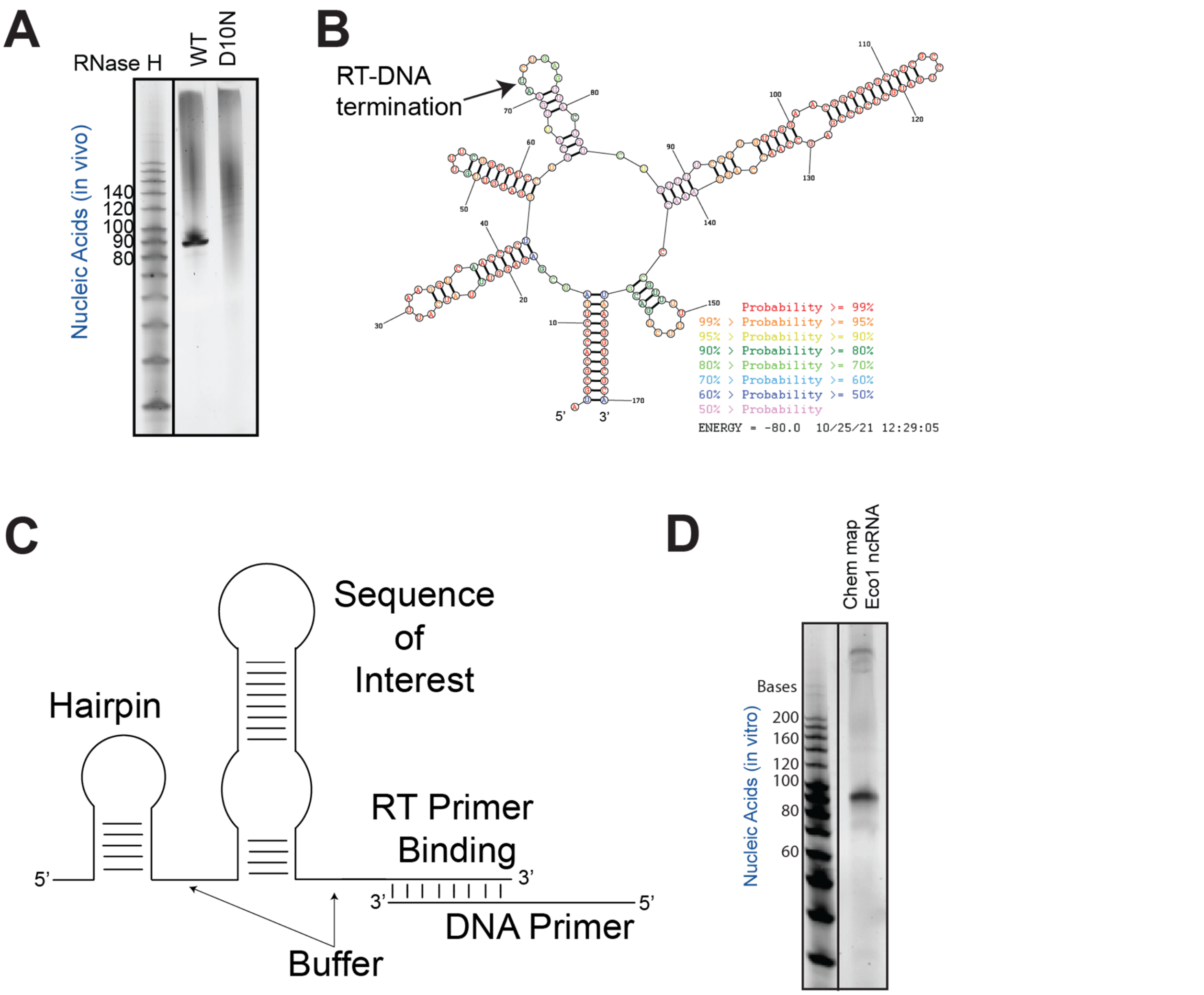

Figure S4. (A) Overexpression of WT RNase H rescues Eco1 RT-DNA length in vivo but RNase H with a mutation in the catalytic amino acid D10 does not rescue length. (B) *In silico* structure prediction of Eco1 ncRNA. (C) Schematic of chemical mapping construct. (D) *In vitro* functional assay using Eco1 ncRNA with added hairpin and RT primer binding sequence demonstrates that construct is functional.

Figure S5

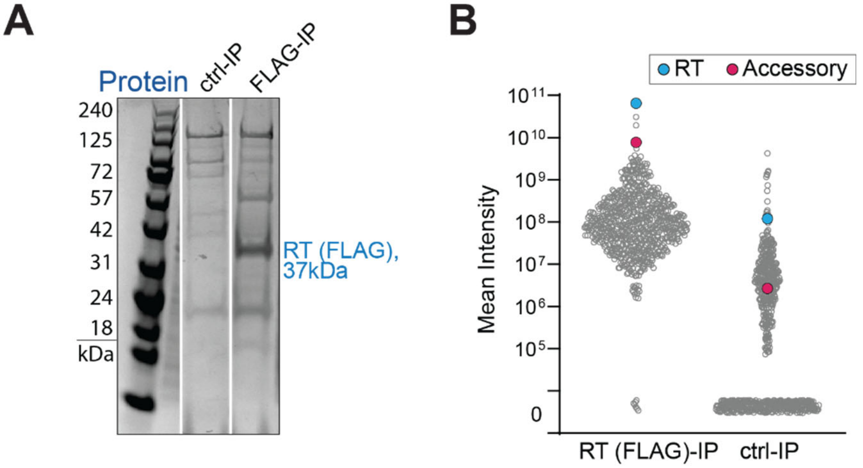

Figure S5: Eco1 accessory protein is enriched when Eco1-RT FLAG immunoprecipitation is performed. (A) SDS-PAGE gel of control-IP (His-tagged retron) vs FLAG-IP after FLAG resin pulldown. Note that the predicted molecular weight of the accessory protein is 35 kDa. (B) Plot of mean intensity in the FLAG-RT pulldown vs the control IP pulldown, highlighting RT (blue) and Accessory (red). Each dot represents an individual protein's mean intensity value across four replicates. The Accessory protein is not overexpressed, thus the high intensity in the FLAG-IP condition suggests either a direct or indirect interaction. The overexpression of the RT accounts for the relatively high mean intensity in the ctrl-IP.
