# Supplementary material for: Retron reverse transcriptase termination and phage defense are dependent on host RNase H1
Source: Nucleic Acids Res. 2022 Mar 16;50(6):3490–504. doi: 10.1093/nar/gkac177 (PMC8989520; doi:10.1093/nar/gkac177)
Supplement: gkac177_Supplemental_Files [file gkac177_supplemental_files.zip › Supplemental_Table_Captions.docx]

**SUPPLEMENTAL TABLE CAPTIONS**

**Supplemental Table 1: Statistical details for experiments in Figures 1, 5, and 6.**

**Supplemental Table 2: Plasmids used in this study.**

**Supplemental Table 3: Bacterial strains used in this study.**

**Supplemental Table 4: Primers and oligonucleotides used in this study.**

**Supplemental Table 5: Additional details of retrons used in this study.**
